# Longitudinal Associations Between Fatalism Dimensions and Different Areas of Health and Well-Being

**DOI:** 10.1093/geroni/igaf122.2707

**Published:** 2025-12-31

**Authors:** Masahiro Toyama, Chloe Hooper

**Affiliations:** Marshall University, Huntington, West Virginia, United States; Marshall University, Huntington, West Virginia, United States

## Abstract

Previous research overall suggested that fatalism, closely related to external locus of control, had negative health implications. However, as older adults tend to have more uncontrollable experiences, fatalism may serve as a protective factor. For example, our previous cross-sectional research for older adults indicated that the fatalism dimension of ineluctable destiny (i.e., beliefs in fate) was associated with lower depression after controlling for helplessness as another fatalism dimension. The present study examined longitudinal associations between fatalism dimensions and different areas of health and well-being to address their directionality. Among 476 participants aged 65 + (M = 70.0, SD = 4.4; 62% female) recruited via an online crowdsourcing platform (Prolific) and included in our previous research (at Time 1 [T1]), 396 completed our six-month follow-up study (at Time 2 [T2]). We analyzed a path analysis model addressing both directions of associations: from five T1 fatalism dimensions to T2 depression, physical functioning, and purpose in life; and from T1 health/well-being measures to T2 fatalism dimensions. For each T2 outcome, its T1/baseline levels and other covariates were controlled for. The results showed good model fit indices with a robust maximum likelihood estimation; ineluctable destiny predicted lower depression over time, while depression did not predict ineluctable destiny and none of the other fatalism dimensions predicted any health/well-being outcomes. These longitudinal findings highlight the positive implications of beliefs in fate for (lower) depression. The present study contributes by adding nuance to the fatalism-aging literature and suggests future research directions (e.g., intervention studies increasing certain fatalistic beliefs).

